# Machine learning identifies a distinct microbiota signature in immune checkpoint inhibitor colitis compared with inflammatory bowel disease

**DOI:** 10.1093/oncolo/oyaf376

**Published:** 2025-11-17

**Authors:** Brigida Barberio, Ilaria Patuzzi, Luisa Bertin, Sonia Facchin, Marianna De Ruvo, Erica Bonazzi, Caterina De Barba, Alessandro Dal Maso, Stefano Frega, Barbara Simionati, Eleonora Sattin, Andrea Buda, Fabiana Zingone, Patrizia Burra, Angelo Paolo Dei Tos, Giulia Pasello, Laura Bonanno, Edoardo Vincenzo Savarino

**Affiliations:** Gastroenterology Unit, Department of Surgery, Oncology and Gastroenterology, University Hospital of Padova, Padova 35100, Italy; Research & Development Division, EuBiome Srl, Via della Croce Rossa 112, Padova, Veneto 35100, Italy; Gastroenterology Unit, Department of Surgery, Oncology and Gastroenterology, University Hospital of Padova, Padova 35100, Italy; Gastroenterology Unit, Department of Surgery, Oncology and Gastroenterology, University Hospital of Padova, Padova 35100, Italy; Gastroenterology Unit, Department of Gastrointestinal Oncological Surgery, S. Maria del Prato Hospital, Feltre 32032, Italy; Gastroenterology Unit, Department of Surgery, Oncology and Gastroenterology, University Hospital of Padova, Padova 35100, Italy; Gastroenterology Unit, Department of Surgery, Oncology and Gastroenterology, University Hospital of Padova, Padova 35100, Italy; Veneto Institute of Oncology IOV-IRCCS, Padova 35100, Italy; Veneto Institute of Oncology IOV-IRCCS, Padova 35100, Italy; Research & Development Division, EuBiome Srl, Via della Croce Rossa 112, Padova, Veneto 35100, Italy; BMR Genomics Via Redipuglia 22, Padova 35100, Italy; Gastroenterology Unit, Department of Gastrointestinal Oncological Surgery, S. Maria del Prato Hospital, Feltre 32032, Italy; Gastroenterology Unit, Department of Surgery, Oncology and Gastroenterology, University Hospital of Padova, Padova 35100, Italy; Gastroenterology Unit, Department of Surgery, Oncology and Gastroenterology, University Hospital of Padova, Padova 35100, Italy; Surgical Pathology and Cytopathology Unit, Department of Medicine-DIMED, University of Padua School of Medicine, Padova 35100, Italy; Department of Surgery, Oncology and Gastroenterology, University of Padova, Padova 35100, Italy; Medical Oncology 2, Istituto Oncologico Veneto IOV IRCCS, Padova 35100, Italy; Department of Surgery, Oncology and Gastroenterology, University of Padova, Padova 35100, Italy; Medical Oncology 2, Istituto Oncologico Veneto IOV IRCCS, Padova 35100, Italy; Gastroenterology Unit, Department of Surgery, Oncology and Gastroenterology, University Hospital of Padova, Padova 35100, Italy

**Keywords:** inflammatory bowel disease, microbiota, ulcerative colitis, Crohn’s disease, immune checkpoint inhibitor colitis, machine learning

## Abstract

**Background:**

Immune checkpoint inhibitors (ICIs) have revolutionized oncology by enhancing antitumor immune responses. However, their use is frequently associated with immune-mediated adverse events, including colitis (ICIs-colitis). This condition shares clinical and histological features with inflammatory bowel diseases (IBD), such as Crohn’s disease (CD) and ulcerative colitis (UC). Emerging evidence highlights the gut microbiota’s role in both ICIs-colitis and IBD pathogenesis. This study aimed to determine whether a distinct microbiota profile, assessed via machine learning, could differentiate ICIs-colitis from IBD and healthy controls (HCs).

**Methods:**

A prospective study was conducted with patients diagnosed with ICIs-colitis, alongside historical cohorts of IBD patients (active and inactive UC/CD) and HCs. Stool samples were analyzed using 16S rRNA gene sequencing. Diversity metrics (alpha and beta) and differential abundance at multiple taxonomic levels were evaluated. Machine learning techniques, including supervised and unsupervised algorithms, were employed to identify microbiota patterns and signatures distinguishing the groups.

**Results:**

Nineteen patients with ICIs-colitis, 40 with UC (20 active, 20 inactive), 34 with CD (14 active, 20 inactive), and 36 HCs were analyzed. Alpha diversity differed between ICI-colitis and UC (*P* = .03) and between ICI-colitis and CD (*P* = .0002), but not versus healthy controls (*P* = .94). Beta diversity showed significant disease-associated clustering among ICI-colitis, UC and CD (PERMANOVA *P* < .001). Differential abundance analyses identified higher *Enhydrobacter* in ICI-colitis versus IBD and higher *Bifidobacterium longum* in UC. Machine-learning approaches (sparse partial least squares discriminant analysis, sPLS-DA, and Random Forest) supported group discrimination.

**Conclusion:**

A unique microbiota signature characterizes ICIs-colitis compared to IBD and HCs. These findings underscore the potential of microbiota profiling and machine learning to aid in diagnosing and managing ICIs-colitis. Future studies should validate these findings in larger, multicenter cohorts, and explore therapeutic implications.

Implications for practice Our findings demonstrate that immune checkpoint inhibitor (ICI)-related colitis harbors a distinct gut microbiota signature compared to inflammatory bowel diseases and healthy controls. This distinction, identified through advanced sequencing and machine learning, highlights the potential of microbiota profiling as a complementary diagnostic tool in oncological practice. For clinicians, integrating microbiota analysis may improve differential diagnosis, avoiding misclassification with ulcerative colitis or Crohn’s disease, and guiding timely management of immune-mediated adverse events. Furthermore, these results open perspectives for microbiota-targeted interventions to prevent or mitigate ICI-colitis, ultimately optimizing both cancer therapy continuity and patient outcomes.

## Introduction

Immune checkpoint inhibitors (ICIs) represent a significant advancement in oncology, targeting key pathways that regulate immune responses. By inhibiting cytotoxic T lymphocyte antigen 4 (CTLA-4) and programmed death 1 (PD-1) or its ligand (PD-L1), these therapies have transformed treatment approaches across various malignancies.^1^^,^^2^ However, their use is accompanied by immune-related adverse events (irAEs), which frequently involve the gastrointestinal tract, manifesting as ICIs-related colitis.^1^^,^^2^ This condition ranges from mild diarrhea to severe colitis, often appearing within weeks of initiating therapy but potentially occurring later or after cessation of treatment.^3^

The mechanisms underlying ICIs-colitis are not fully understood. Dysregulation of the gut microbiota has been implicated, with ICIs potentially disrupting the microbiota-intestinal barrier equilibrium.^3^ This disruption may involve the induction of apoptosis in intestinal epithelial cells, leading to increased barrier permeability and microbiota alterations.^4^ These changes can trigger innate immune responses and activate self-reactive immune cells.^5^^,^^6^ Evidence suggests reduced microbiota diversity in ICIs-colitis, including alterations in specific taxa such as decreased *Bacteroides fragilis* and increased *Faecalibacterium prausnitzii.*^7^

Notably, ICIs-colitis exhibits similarities to inflammatory bowel disease (IBD), including Crohn’s disease (CD) and ulcerative colitis (UC), in terms of clinical presentation and histopathological findings.^8^ These overlaps can complicate differential diagnosis. Particularly, endoscopic findings are nonspecific, since normal mucosa, mucosal edema, erythema, erosions, loss of vascular pattern, and superficial or deep mucosal ulcerations, either in a patchy or continuous fashion have been shown in patients with ICIs-colitis.^9^ Even endoscopically normal mucosa should be biopsied to exclude microscopic inflammation.^10^ In fact, histological features of ICIs-colitis vary and may show changes of acute colitis, chronic colitis (IBD-like), acute and chronic colitis, or microscopic colitis.^9^

Given the pivotal role of the gut microbiota in the pathogenesis of both ICIs-colitis and IBD^11^^,^^12^ our study aimed to investigate whether distinct gut microbiota signatures can differentiate ICIs-colitis from IBD and healthy controls (HCs). Leveraging 16S rRNA gene sequencing and machine learning approaches, we sought to characterize these microbiota patterns and explore their potential clinical applications.

## Methods

### Study populations, sample, and data collection

This prospective study included patients diagnosed with ICIs-colitis at University Hospital of Padova, Italy, from January 2021 to December 2023. Historical cohorts of IBD patients (both active and inactive UC/CD) and HCs were included for comparison.^13^^,^^14^ Inclusion and exclusion criteria are reported in Supplementary Table S1.

For each patient, stool samples were collected within 24 h of defecation, stored at 4 °C, and transferred to the laboratory for preservation in e-Nat kits (Copan Italia S.p.A.) at −20 °C. DNA was extracted, and the V3-V4 region of the 16S rRNA gene was amplified for sequencing using Illumina MiSeq technology. Fecal samples of HCs were collected at home on the evening before, or the morning of, its delivery to our laboratory, again within 24 h of defecation. For patients with IBD with moderately-to-severely active disease, fecal samples were collected at home if they were not hospitalized or were collected within 24 h of hospitalization in the case of hospitalized patients. Fecal samples from patients with ICIs-colitis were collected at the time of colitis diagnosis, after a median of 3 months (IQR: 2-6) from ICI initiation. Samples were obtained either during ongoing immunotherapy or immediately after ICI discontinuation due to colitis, and always before starting any steroids, immunosuppressants, or biologic therapy.

The following data were recorded for each patient with IBD at baseline: age, gender, age at diagnosis, disease duration, disease location and extent, previous biological treatments, and presence of extraintestinal manifestations. Clinical activity was measured by using partial Mayo (p-Mayo) Score and Harvey–Bradshaw Index (HBI) for UC and CD, respectively. According to medical literature, clinical activity was classified into remission/mild activity, moderate to severe according to the following values of pMayo for UC: 0-4 remission/mild disease, 5-9 moderate to severe disease.^15^ Conversely, the following values of HBI were used for CD patients: <7 remission/mild disease, >7 moderate to severe disease.^16^ In patients with CD and UC, we also evaluated biochemical activity dosing fecal calprotectin with a value > 250 µg/g indicating an active disease. For the purpose of the study, all endoscopic examinations were performed within 3 to 5 days of stool collection.

### Sample processing and sequencing

For the microbiota analysis, the stool samples were preserved in eNAT kits and stored at −20 °C until analysis. Sequencing protocol was performed at BMR Genomics srl. Briefly: V3–V4 regions of 16S rRNA gene were amplified using the primers Pro341F: 5′-CCTACGGGNBGCASCAG-3′ and Pro805R: Rev 5′-GACTACNVGGGTATCTAATCC-3′.^17^ Primers were modified with forward overhang: 5′-TCGTCGGCAGC GTCAGATGTGTATAAGAGACAG [locus-specific sequence]-3′ and with reverse overhang: 5′-GTCTCGTGGGCTCGGAGATGTGTA TAAGAGACAG [locus-specific sequence]-3′ necessary for dual-index library preparation, following Illumina protocol https://web.uri.edu/gsc/files/16s-metagenomic-library-prep-guide-15044223-b.pdf. Samples were normalized, pooled, and run on Illumina MiSeq with 2 × 300 bp approach.

### Bioinformatic analysis and statistics

The raw reads underwent a filtering procedure performed within QIIME2 analysis framework (version 2020.8).^18^ The cutadapt plugin was utilized to remove primers, followed by quality filtering, denoising, and chimera detection using the DADA2 plugin. Alpha diversity was assessed on rarefied datasets using the Richness, Shannon, and Pielou indices, with a rarefaction threshold set at 31 556. Beta diversity calculations were performed on normalized counts using multiple distance metrics, including Bray–Curtis, Jaccard, Canberra, Weighted, and Unweighted UniFrac, with GMPR applied for normalization.^19^ The diversity assessments were conducted in R (version 4.1.0) utilizing the DiversitySeq package, and statistical analyses of alpha diversity indices between groups were performed using either the Kruskal-Wallis or ANOVA tests, depending on data normality.

To examine microbiota composition variations among disease groups, a permutational analysis of variance (PERMANOVA) was applied to Bray–Curtis dissimilarities, utilizing the vegan and ecole packages. The MaAsLin2 package facilitated differential abundance analysis across all taxonomic levels. Supervised and unsupervised machine learning approaches were employed on normalized datasets to evaluate potential sample clustering and classification while identifying the most relevant taxa for group differentiation. Accordingly, nonmetric multidimensional scaling (NMDS) was conducted based on Bray–Curtis distances, alongside random forest classification and sparse partial least squares discriminant analysis (sPLS-DA). Machine learning methodologies were implemented in R using the stats, phyloseq, randomForest, and mixOmics packages. The sPLS-DA model highlighted key amplicon sequence variants (ASVs), assigning loading values indicative of their role in distinguishing sample groups.

Finally, demographic and clinical data were compared between individuals with inactive (remission/mild disease) and active (moderate to severe) UC or CD, employing SPSS for Windows (version 24.0, SPSS Inc., Chicago, IL, USA). Continuous variables were expressed as medians with ranges, while categorical data were summarized as frequencies and percentages.

## Results

The study included 19 patients with ICIs-colitis, 40 UC patients (20 patients with active UC and 20 with inactive UC), 34 patients with CD (14 patients with active CD and 20 with inactive CD), and 36 HCs at IBD Unit of Padova University Hospital and at Veneto Oncological Institute (Padova, Italy).

Fifteen patients with ICIs colitis had non-small-cell lung carcinoma (NSCLC) and four a melanoma skin cancer. Regarding the ICIs therapy administered, 15 were treated with pembrolizumab (anti-PD1) and 4 with nivolumab (anti-PD1). Moreover, looking at the histological features of patients with ICIs colitis, 10 had an IBD-like colitis, 9 had a microscopic colitis.

Clinical and demographic data are summarized in Tables 1 and 2. Included HCs had a median age of 37 years, with a male to female ratio of 1:1.

**Table 1.** Clinical and demographic characteristics of patients enrolled in the study, encompassing those diagnosed with Crohn’s disease, ulcerative colitis.

		Crohn’s disease		Ulcerative colitis
		*n* = 34		*n* = 40
**Age at inclusion (years)**	Mean (SD)	44 (15)	Age at inclusion (years)	48 (14)
**Smokers**	*n* (%)	2 (5.9%)	Smokers	3 (7.5%)
**Female**	*n* (%)	11 (32.3%)	Female	15 (37.5%)
**BMI**	Median (IQR)	22 (20.94-25.98)	BMI	23.3 (37.7-55.2)
**Disease activity**			Disease activity	
**SES-CD score**	Median (IQR)	4 (1.5-7)	Mayo endoscopic subscore	2 (0.75-2.25)
**HBI-score**	Median (IQR)	1 (0-5)	Mayo partial score	2 (0.5-4.5)
**Fecal calprotectin (mg/kg)**	Median (IQR)	171 (57-550)	Fecal calprotectin (mg/kg)	203 (70-1101)
**Disease location**			Disease location	6 (15.0%)
** Ileum**	*n* (%)	6 (17.6%)	Ulcerative Proctitis	16 (4.0%)
** Colon**	*n* (%)	8 (23.5%)	Left-sided	21 (52.5%)
** Ileocolonic**	*n* (%)	22 (64.7%)	Extensive	1 (2.5%)
** Additional upper GI**	*n* (%)	9 (26.4%)	-	
**Disease behavior**			-	
**Inflammatory**	*n* (%)	Biologic therapy	-	
** Stricturing**	*n* (%)	Immunosuppressors	-	
** Penetrating**	*n* (%)	Steroids	-	
** Perianal disease**	*n* (%)	5-ASA	-	
**Prior intestinal resection**	*n* (%)	11 (32.3%)	-	
**Prior perianal fistula surgical intervention**	*n* (%)	3 (8.82%)	-	
**Current treatment**			Current treatment	
**Biologic therapy**	*n* (%)	20 (5.88%)	Biologic therapy	16 (40.0%)
**Immunosuppressors**	*n* (%)	5 (14.7%)	Immunosuppressors	8 (20.0%)
**Steroids**	*n* (%)	1 (2.94%)	Steroids	6 (15%)
**5-ASA**	*n* (%)	27 (79.4%)	5-ASA	35 (87.5%)

**Table 2.** Clinical and demographic characteristics of patients enrolled in the study, encompassing those diagnosed with ICIs-colitis.

		*n* = 19
**Age at inclusion (years)**	Mean (SD)	70 (60-74.5)
**Smokers**	*n* (%)	2 (10.5%)
**Female**	*n* (%)	13 (68%)
**BMI**	Median (IQR)	23.8 (21.6-26.1)
**Charlson Comorbidity Index**	Median (IQR)	8 (6-9)
**Pre existing IBD**	*n* (%)	1 (5.3%)
**Previous diagnosis of immune-mediated or autoimmune disorders**	*n* (%)	6 (31.5%)
**Tumour - Type**		
** Melanoma**	*n* (%)	2 (10.5%)
** Lung adenocarcinoma**	*n* (%)	11 (57.9%)
** Squamous lung carcinoma**	*n* (%)	4 (21.1%)
** Colic adenocarcinoma**	*n* (%)	1 (5.3%)
** Pleural mesothelioma**	*n* (%)	1 (5.3%)
**Tumour - Localisation**		
** Lung**	*n* (%)	15 (78.9%)
** Colon**	*n* (%)	1 (5.3%)
** Pleura**	*n* (%)	1 (5.3%)
** Skin**	*n* (%)	2 (10.5%)
**Tumour staging**		
** T1**	*n* (%)	0 (0%)
** T2**	*n* (%)	3 (15.8%)
** T3**	*n* (%)	10 (52.6%)
** T4**	*n* (%)	6 (31.6%)
** Nx**	*n* (%)	2 (10.5%)
** N0**	*n* (%)	2 (10.5%)
** N1**	*n* (%)	5 (26.3%)
** N2**	*n* (%)	7 (36.8%)
** N3**	*n* (%)	3 (15.8%)
** M0**	*n* (%)	8 (42.1%)
** M1**	*n* (%)	11 (57.9%)
**Immune checkpoint inhibitors type**		
** Ipilimumab**	*n* (%)	0 (0%)
** Pembrolizumab**	*n* (%)	16
** Nivolumab**	*n* (%)	3 (15.8%)
** Atezolizumab**	*n* (%)	0 (0%)
** Avelumab**	*n* (%)	0 (0%)
** Durvalumab**	*n* (%)	0 (0%)
** Cemiplimab**	*n* (%)	0 (0%)
**Oncological therapy other than ICI**		
** Surgery**	*n* (%)	4 (21.1%)
** Radiotherapy**	*n* (%)	4 (21.1%)
** Chemotherapy**	*n* (%)	6 (31.6%)
**ICI - immune-mediated adverse reaction**		
** Hypophysitis**	*n* (%)	0 (0%)
** Autoimmune thyroid disease**	*n* (%)	2 (10.5%)
** Autoimmune hepatitis**	*n* (%)	0 (0%)
** Adrenalitis**	*n* (%)	1 (5.3%)
** Skin changes**	*n* (%)	1 (5.3%)
** Interstitial lung disease**	*n* (%)	0 (0%)
** Nephritis**	*n* (%)	5 (26.3%)
**ICI - nonimmune-mediated adverse reaction**		
** Pruritus**	*n* (%)	4 (21.1%)
** Fatigue**	*n* (%)	1 (5.3%)
** Neutropenia**	*n* (%)	
**Time of symptom onset relative to the initiation of ICIs therapy**	Median (IQR)	1 (5.3%)
**ICI colitis characteristics at baseline**		
** Diarrhoea - severity grading by CTCAE**	Median (IQR)	2 (2-3)
** Colitis - severity grading by CTCAE**	Median (IQR)	2 (1-2)
** Fecal calprotectin levels**	Median (IQR)	958 (496-1886)
**Macroscopic findings at colonoscopy**		
** Normal appearance**	*n* (%)	7 (36.8%)
** Diffuse nonulcerative inflammation**	*n* (%)	6 (31.6%)
** Mucosal ulcerations**	*n* (%)	4 (21.1%)
** UC-like pattern**	*n* (%)	8 (41.1%)
** CD-like pattern**	*n* (%)	3 (15.8%)
**Disease extension at index colonoscopy**		
** Normal appearance**	*n* (%)	7 (36.8%)
** Terminal ileum**	*n* (%)	0 (0%)
** Ileocolic**	*n* (%)	1 (5.3%)
** Colic**	*n* (%)	11 (57.4%)
**Histologic findings at index colonoscopy**		
** Normal**	*n* (%)	0 (0%)
** Increased intraepithelial Lymphocytes**	*n* (%)	7 (36.8%)
** Collagenous band**	*n* (%)	9 (47.4%)
** Cryptitis**	*n* (%)	1 (5.3%)
** Crypt abscesses**	*n* (%)	2 (10.5%)
** Apoptosis**	*n* (%)	5 (26.3%)
** Gland architecture abnormalities**	*n* (%)	7 (36.8%)
**ICIs-colitis treatment**		
** Biologic therapy**	*n* (%)	1 (5.3%)
** Immunosuppressors**	*n* (%)	0 (0%)
** Systemic steroids**	*n* (%)	14 (73.7%)
** Topical steroids**	*n* (%)	7 (36.8%)
** 5-ASA**	*n* (%)	4 (21.1%)
**ICIs discontinued because of colitis**		15 (78.9%)
**Outcomes after ICIs-colitis**		
** ICIs resumption/continuation**	*n* (%)	9 (47.4%)
** Change of therapy from ICIs**	*n* (%)	13 (68.4%)
** Death due to cancer progression or complication**	*n* (%)	1 (5.3%)

### Preprocessing: from reads to annotated amplicon sequence variant table

A total of 22 460 542 paired end reads (11 230 271 from forward, 11 230 271 from reverse FASTQ files; mean: 87 056.36; SD: 35 041.06) were obtained from the sequencing procedure. After the filtering, denoising and chimera checking steps, a total of 5 922 344 (mean: 45 909.64; SD: 14 606.14) non-chimeric reads were retained. The resulting amplicon sequence variant (ASV) table collected 4970 ASVs belonging to 14 phyla, 30 classes, 54 orders, 106 families, 245 genera, and 367 species.

### Metataxonomics results

**Figure 1.**
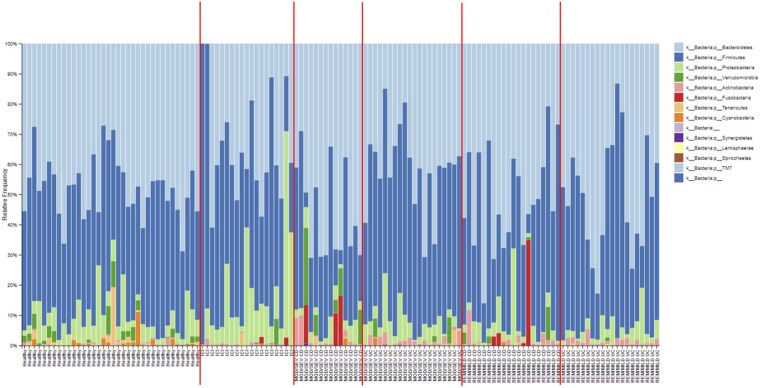
Microbiota composition (Phylum level) in four different groups of patients (Healthy controls, UC, CD, ICIs-colitis). Microbiota composition at phylum level across subgroups. Subpanels are labeled as: Healthy controls, UC (remission/mild and moderate/severe), CD (remission/mild and moderate/severe), and ICI-colitis. Bars represent relative abundance (%). Error bars, where present, indicate interquartile range. Abbreviations: MOD/SEV CD: moderate to severe Crohn’s disease; MOD/SEV UC: moderate to severe ulcerative colitis; REM/MILD CD: remission/mild Crohn’s disease; REM/MILD UC: remission/mild ulcerative colitis; ICI: Immune checkpoint inhibitors (meaning ICIs-colitis).

**Figure 2.**
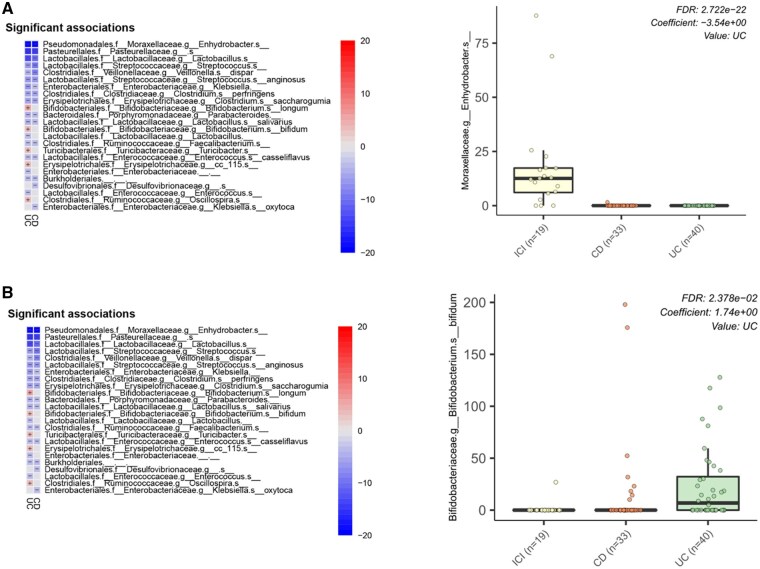
Microbiota composition: different abundant species (respect to ICIs-colitis). Differentially abundant taxa comparing ICIs-colitis versus IBD. (A) At genus levels. (B) At species levels. Left panels show log2 fold change (unitless); right panels show standardized model coefficients (*β*) from MaAsLin2. The *y*-axis lists taxa. Boxes depict interquartile ranges with the median; whiskers extend to 1.5× IQR; black dots represent outliers. *P*-values are FDR-adjusted. Abbreviations: CD: Crohn’s disease; UC: moderate to severe ulcerative colitis; ICI: Immune checkpoint inhibitors (meaning ICIs-colitis).

At phylum level, we found high levels of *Tenericutes* in HC, while *Proteobacteria* were more abundant in patients with ICIs-colitis. *Actinobacteria* was significantly more abundant in patients with IBD and reduced in patients with ICIs-colitis (Figure 1). Performing a differential abundance analysis, interestingly, at genus levels, we found very high levels of *Enhydrobacter* (unknown species) in ICIs-colitis compared to patients with IBD (FDR—false discovery rate: 2.722 e−22) (Figure 2A). While at species levels, high levels of *Bifidobacterium longum* in patients with UC were observed (FDR: 2.378 e−02) (Figure 2B). Patients with ICIs-colitis included those with microscopic features IBD-like and those with microscopic colitis; when we compared patients with UC with only those with IBD-like colitis, at species levels, we found that high levels of *Enhydrobacter* (FDR: 6.571 e−08) and of *Streptococcus* (unknown species) (FDR: 9.245 e−03) in patients with IBD-like ICIs-colitis compared to those with UC (Supplementary Figure S1).

Exploratory analyses stratifying patients by ICI agent (pembrolizumab vs nivolumab) and by underlying malignancy (NSCLC vs melanoma) did not reveal significant differences in microbiota composition, although these analyses were underpowered.

### Alpha diversity analysis

**Figure 3.**
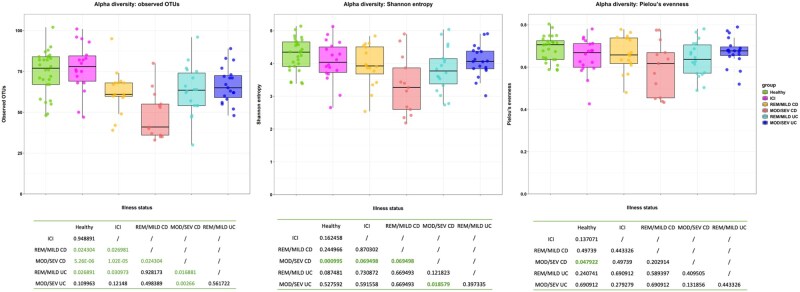
Alpha diversity analysis results for richness, Shannon, and Pielou indices. Alpha diversity metrics: Observed OTUs richness (count), Shannon diversity, Pielou evenness. All *y*-axes start at 0. Boxes depict IQR with median; whiskers extend to 1.5× IQR. Group abbreviations: MOD/SEV CD, MOD/SEV UC, REM/MILD CD, REM/MILD UC, and ICI-colitis. Abbreviations: MOD/SEV CD: moderate to severe Crohn’s disease; MOD/SEV UC: moderate to severe ulcerative colitis; REM/MILD CD: remission/mild Crohn’s disease; REM/MILD UC: remission/mild ulcerative colitis; ICI: Immune checkpoint inhibitors (meaning ICIs-colitis).

Alpha diversity analysis (ASV richness, Shannon index, and Pielou index) revealed distinct patterns among groups (Figure 3), observing a statistically significant alpha diversity (species richness) when we compared patients with ICIs-colitis with patients with UC (*P* = .03) and CD (*P* = .0002).

In addition, alpha diversity results (ASV richness, Shannon index, and Pielou index) in patients with ICIs-colitis and those with all patients with UC and with CD (both active and inactive pooled together) are reported in Supplementary Figure S2. Alpha diversity was not significantly different between patients with ICIs-colitis and HCs (*P* = .94), patients with ICIs-colitis and patients with active UC (*P* = .12) and between patients with HCs and patients with active UC (*P* = .11). While, alpha diversity was statistically significant between patients with ICIs-colitis and patients with inactive UC, inactive CD and active CD (*P* = .03, *P* = .03, and *P* < .0001, respectively); it was also statistically significant between HCs and patients with inactive UC, inactive CD and active CD (*P* = .03, *P* = .02, and *P* < .0001, respectively). Even when we evaluated alpha diversity in patients with UC compared with those with IBD-like ICIs colitis we found that Pielou and Shannon indices were statistically significant (*P* = .01 and *P* = .03, respectively) but not the ASV richness (*P* = .90; Supplementary Figure S3).

### Nonmetric multidimensional scaling

**Figure 4.**
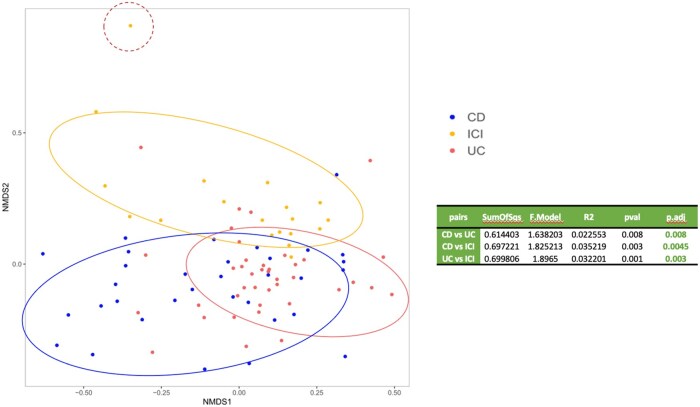
Nonmetric multidimensional scaling (NMDS) plot of beta diversity (Bray–Curtis distance matrix). Nonmetric multidimensional scaling (NMDS) based on Bray–Curtis dissimilarities. Each point represents one sample; colors denote clinical status (ICI-colitis, UC, CD). Ellipses indicate 95% confidence regions; the dashed circle highlights an ICI outlier. The table reports pairwise PERMANOVA results: *SumOfSqs* (sum of squares for the group factor), F. Model (between- vs within-group variance ratio), *R*^2^ (variance explained), and *P*-values with multiple-testing adjustment (*P*.adj).

Following the construction of the ASV table, a graphical ordination analysis was conducted using PERMANOVA to evaluate differences based on the Bray–Curtis dissimilarity metric (Figure 4). Beta-diversity analysis revealed a significant disease-associated clustering across the three groups (*P* < .001), with one ICIs-colitis sample positioned at the greatest distance from both CD and UC patients. Notably, the aforementioned five individuals with active UC, whose microbiota composition markedly differed from the rest of their group, were distinctly separated within the analysis.

Additional beta-diversity comparisons based on Bray–Curtis distances, differentiating ICIs-colitis from active and inactive UC and CD, are presented in Supplementary Figure S4. Moreover, a separate beta-diversity evaluation comparing IBD-like ICIs-colitis with UC patients is available in Supplementary Figure S5.

### sPLS-DA and random forest analyses

**Figure 5.**
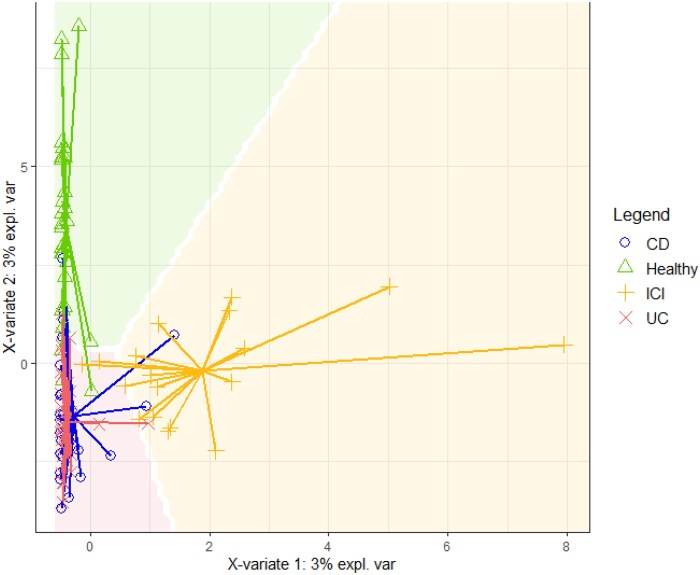
Sparse partial least squares discriminant analysis (SPLS-DA), a machine learning-supervised approach using all ASVs. sPLS-DA using the full ASV table. The first two latent components (X-variates 1 and 2) are shown; although they explain only a small proportion of total variance (≈6% cumulatively), sPLS-DA is optimized for classification rather than variance capture and achieved robust group separation. See Supplementary Figure S6 for Random Forest performance. Abbreviations: CD: Crohn’s disease; UC: moderate to severe ulcerative colitis; ICI: Immune checkpoint inhibitors (meaning ICIs-colitis).

The sPLS-DA analysis allowed us to observe a very good class prediction and group separation using the complete information (full ASV table) (Figure 5). Interestingly, the majority of the features selected by the sPLS-DA algorithm were the ones marked as differentially abundant among groups (IBD, HC, ICIs-colitis). The host trait predictive potential of the microbiota was also confirmed by a Random Forest analysis (Supplementary Figure S6).

## Discussion

Immune-mediated colitis remains a prevalent gastrointestinal complication associated with immune checkpoint inhibitor (ICI) therapy, significantly impacting its utility and management strategies. This condition exhibits clinical and pathological similarities to inflammatory bowel diseases (IBD), particularly Crohn’s disease (CD) and ulcerative colitis (UC), posing challenges in differentiating between these disorders.^20^ Moreover, the gut microbiota is increasingly recognized as a critical factor influencing the development and progression of both ICI-related colitis and IBD.^20^ This study leveraged machine learning techniques to identify unique microbiota patterns associated with ICI-colitis, underscoring their potential in improving diagnostic precision and guiding therapeutic interventions.

Our findings highlighted significant differences in microbial diversity and composition across groups. Alpha diversity analyses revealed variations in species richness, Shannon index, and Pielou index among ICI-colitis, UC, and CD. Statistically significant differences in species richness were found between ICIs-colitis and UC (*P* = .03) as well as CD (*P* = .0002). While no significant differences were found between ICIs-colitis and HCs (*P* = .94). Considering the disease activity in IBD, no significant differences were found between ICIs-colitis and active UC (*P* = .12), while significant differences were observed between ICIs-colitis and inactive UC, inactive CD, and active CD (*P* = .03, *P* = .03, and *P* < .0001, respectively), as well as between HCs and inactive UC, inactive CD, and active CD (*P* = .03, *P* = .02, and *P* < .0001, respectively). Comparing IBD-like ICIs-colitis and UC, significant differences were noted in the Pielou index (*P* = .0152) and Shannon index (*P* = .0349), suggesting variations in microbial community structure. At the phylum level, our data revealed an increased abundance of *Proteobacteria* in ICI-colitis, while *Actinobacteria* levels were reduced compared to IBD. Healthy controls demonstrated high levels of *Tenericutes*. Differential abundance analyses identified *Enhydrobacter* as markedly elevated in ICI-colitis compared to IBD. Furthermore, UC was characterized by an increased abundance of *Bifidobacterium longum*, while patients with ICI-colitis exhibiting IBD-like histology had significantly higher levels of Streptococcus (unknown species) and *Enhydrobacter* compared to UC. These microbial variations were further validated using sPLS-DA, which achieved robust classification accuracy and identified key taxa contributing to group differentiation. Although the first two components of sPLS-DA explained only a small percentage of total variance (≈6% cumulatively), the model is optimized for classification rather than variance capture, and it still achieved robust separation among groups.

Accumulating evidence from prior research supports that the gut microbiota signature has a strong link with irAEs. For instance, in a study by Usyk et al. of advanced-stage melanoma patients undergoing ICIs, stool samples were collected before, during, and after the treatment. Two natural gut microbiome clusters with distinct profiles were identified, and patients with a high proportion of *Bacteroides dorei* in gut microbiota had higher risk of irAE, while the *Bacteroides vulgatus* was identified as a specific dominance strain in the low-risk cluster.^21^ Moreover, Liu et al. demonstrated that patients with NSCLC with severe diarrhea ICIs-related had higher levels of *Stenotrophomonas* and *Streptococcus* compared with patients without irAEs or with mild diarrhea.^22^ Similarly, Chaput et al. collected fecal samples from 26 metastatic melanoma patients before the ICI therapy and, according to the characteristics of baseline microbiota composition, patients were divided into three clusters (A, B, C). Patients in cluster A with a high proportion of *Faecalibacterium* and other *Firmicutes* in the microbiota composition, were prone to develop colitis, while patients without colitis had more Bacteroidetes (Cluster B).^23^

In our study, including mainly patients with NSCLC, we did not match them with patients without colitis, because our main aim was to evaluate the differences in microbiota profile between patients with a ICIs-colitis and patients with a chronic colitis, such as IBD. To our knowledge, this is the first study that focused on these differences. In patients with ICIs-colitis we found a higher abundance of *Prevotella* at phylum level and significantly higher levels of *Enhydrobacter* at genus levels.

Despite the strengths of our approach, including the application of advanced machine learning algorithms and a well-characterized patient cohort, some limitations warrant discussion. First, the relatively small sample size may limit the generalizability of our findings. However, we mitigated this by ensuring homogeneity within the study population and controlling for confounding factors such as ongoing treatments. Second, the single-center design underscores the need for validation in multicenter cohorts to enhance the reproducibility of our results. Third, the cross-sectional nature of the study precludes longitudinal assessments of microbiota dynamics during disease progression or treatment. Additionally, our analysis was restricted to fecal microbiota, without evaluating mucosa-associated microbiota, which may provide complementary insights. Moreover, we did not collect systematic information on patients’ dietary habits, which may influence microbiota composition and represent a potential confounding factor. Future studies should integrate dietary data to better contextualize microbiota findings in ICI-colitis. Additionally, our cohort included only patients treated with anti-PD1 agents, with no representation of anti-CTLA-4 or combination regimens; therefore, the generalizability of our findings to other ICI strategies is limited. Finally, given the modest size of the ICIs-colitis cohort, we did not conduct stratified analyses by ICI agent or underlying cancer type, which limits subgroup-specific inferences.

However, despite these limitations, our findings offer significant implications for clinical practice. Identifying a distinct microbiota signature in ICI-colitis highlights the potential of microbiota profiling as a diagnostic tool. Furthermore, machine learning-based approaches, such as sPLS-DA, demonstrate the feasibility of integrating microbiota data into diagnostic workflows to distinguish ICI-colitis from IBD with high accuracy. Future research should aim to validate these findings in larger, multicenter studies and explore the therapeutic potential of microbiota modulation in managing ICI-related colitis and other irAEs.

From a clinical perspective, microbiota profiling may help refine the differential diagnosis between ICI-colitis and IBD and could eventually guide personalized therapeutic strategies. Future longitudinal studies are warranted to assess microbiota dynamics during ICI therapy and colitis treatment, as well as the impact of interventions such as antibiotics, probiotics, dietary modifications, and fecal microbiota transplantation.

In conclusion, this study provides novel insights into the microbiota alterations associated with ICI-colitis, emphasizing the utility of machine learning in advancing our understanding of this condition. By identifying specific microbial signatures, we pave the way for improved diagnostic and therapeutic strategies, ultimately enhancing the management of patients undergoing ICI therapy.

## Supplementary Material

oyaf376_Supplementary_Data

## Data Availability

The data underlying this study is available within the manuscript and supplementary materials.
