# Supplementary material for: Machine learning identifies a distinct microbiota signature in immune checkpoint inhibitor colitis compared with inflammatory bowel disease
Source: Oncologist. 2025 Nov 17;30(12):oyaf376. doi: 10.1093/oncolo/oyaf376 (PMC12687588; doi:10.1093/oncolo/oyaf376)
Supplement: oyaf376_Supplementary_Data [file oyaf376_supplementary_data.zip › Supplementary Material.docx]

**Supplementary Table 1. Inclusion and Exclusion Criteria**

| Healthy Controls: Inclusion Criteria  The Healthy controls enrolled were subjects of both sexes, aged ≥18 years, of Italian nationality, with no relatives with IBD and/or hereditary cancer/syndrome, and who did not present evidence of illness on the basis of the anamnestic data collected. |
| --- |
| Patients with ICIs-colitis: Inclusion criteria  Patients with any type of cancer who assumed ICIs (anti-PD1) for their cancers and who developed a clinical and eventually endoscopic colitis related to treatment.  Stool samples were collected before starting treatment with probiotics, prebiotics, steroids or biological drugs. |
| Patients with Inactive Crohn’s Disease or Inactive Ulcerative colitis: Inclusion Criteria   - - - - Patients with inactive disease were subjects of both sexes.       - Inactive disease was assessed by clinical evaluation and endoscopy with biopsies.       - Patients with UC with a total Mayo score <3 or partial Mayo score <2, with Mayo endoscopic subscore of 0-1 and inactive histological disease (according to Robarts index), and with fecal calprotectin <250 µg/g.       - Patients with CD with HBI <7, with SES-CD≤2 and inactive histological disease, and with fecal calprotectin <250 µg/g. |
| Patients with Active Crohn’s Disease or Active Ulcerative colitis: Inclusion Criteria   - - - - Patients with active disease were subjects of both sexes       - Active disease was assessed by clinical evaluation and endoscopy with biopsies       - Patients with UC with total Mayo score >10 or partial Mayo score >7, with Mayo endoscopic subscore of 3 and active histological disease (according to Robarts index), and with fecal calprotectin ≥250 µg/g.       - Patients with CD with HBI >7, with SES-CD ≥ 7 and active histological disease, and with fecal calprotectin ≥250 µg/g. |
| IBD Patients (Both Active and Inactive): Exclusion Criteria   - - - - Under 18 years of age       - Pregnancy       - Prior proctocolectomy       - Presence of stoma       - Concomitant treatment with antibiotics, prebiotics, steroids, biological therapies, thiopurines or methotrexate, or anticoagulant drugs. Only treatment with mesalazine was allowed. |

**Supplementary Figure 1. Microbiota composition: Different Abundant Species (UC vs IBD-like ICIs-colitis)**

**
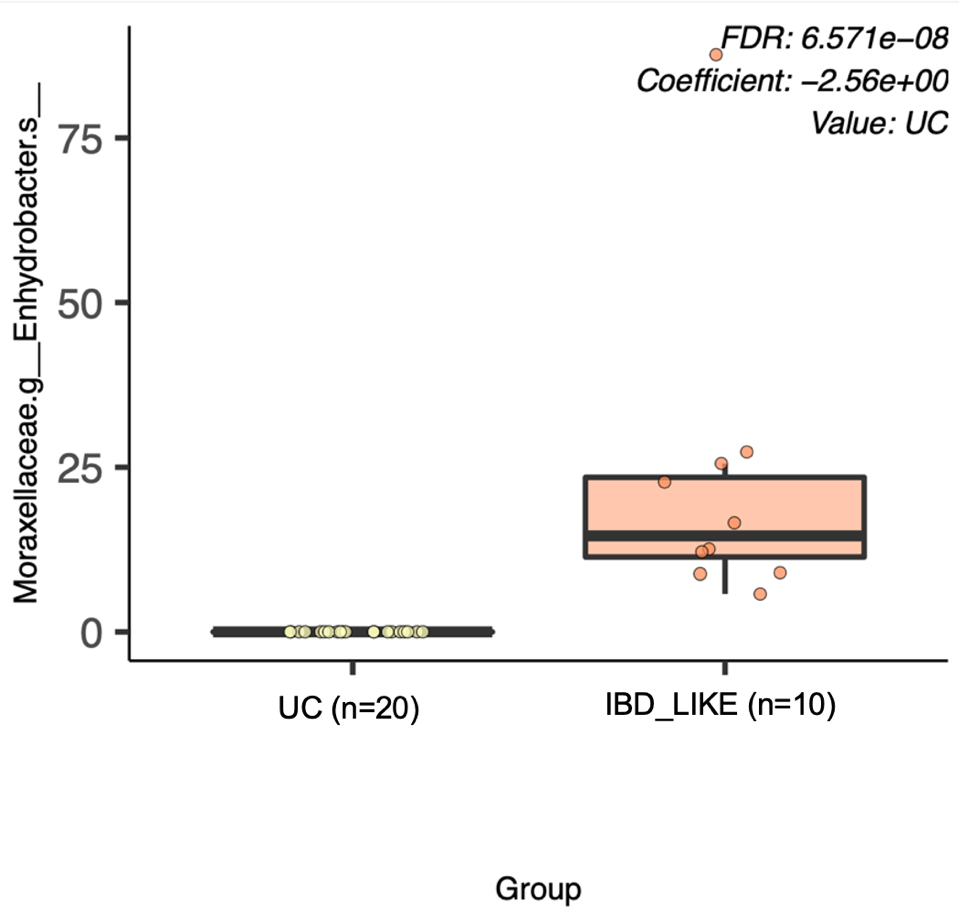
**

**
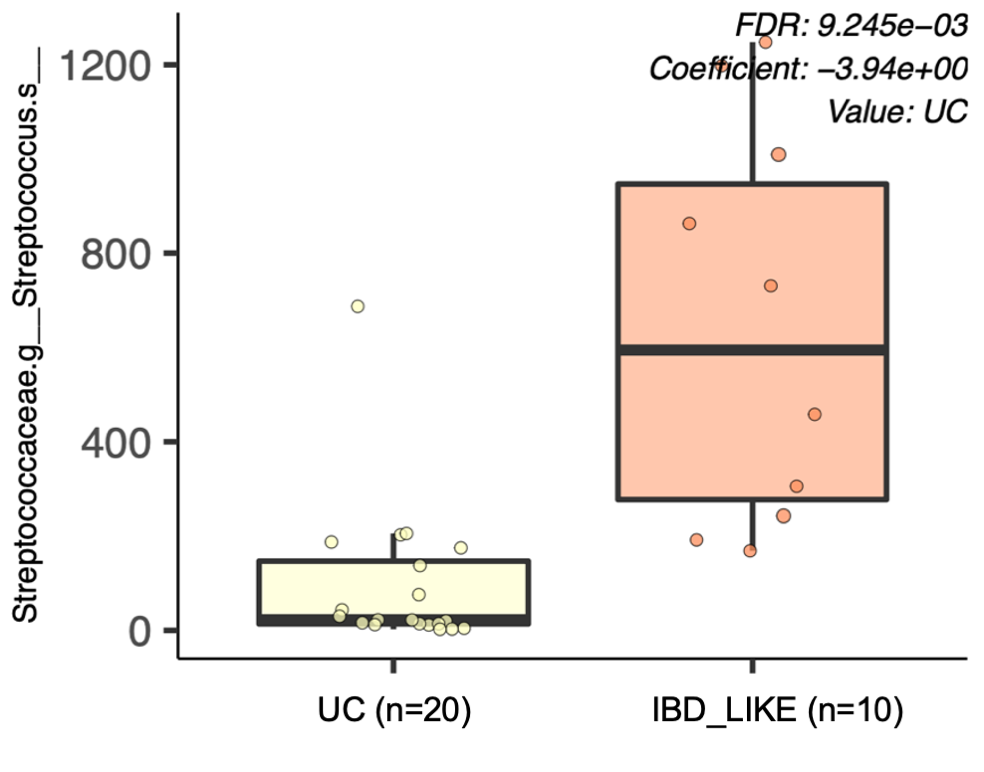
**

**Supplementary Figure 2. Alpha diversity analysis results for richness (A), Shannon (B), and Pielou (C) indices in patients with UC, CD and those with ICIs-colitis.**

**A.**

**
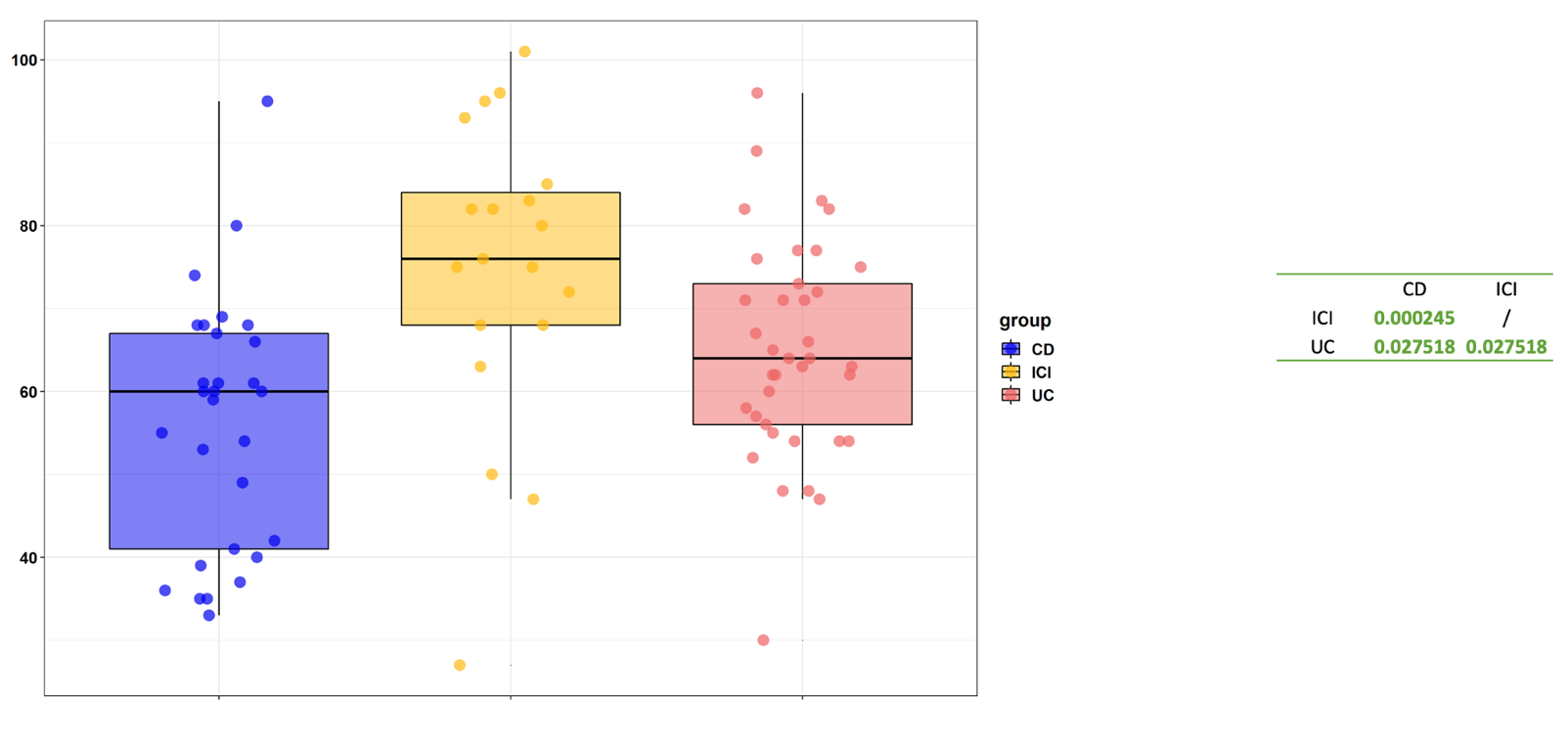
**

**B.**


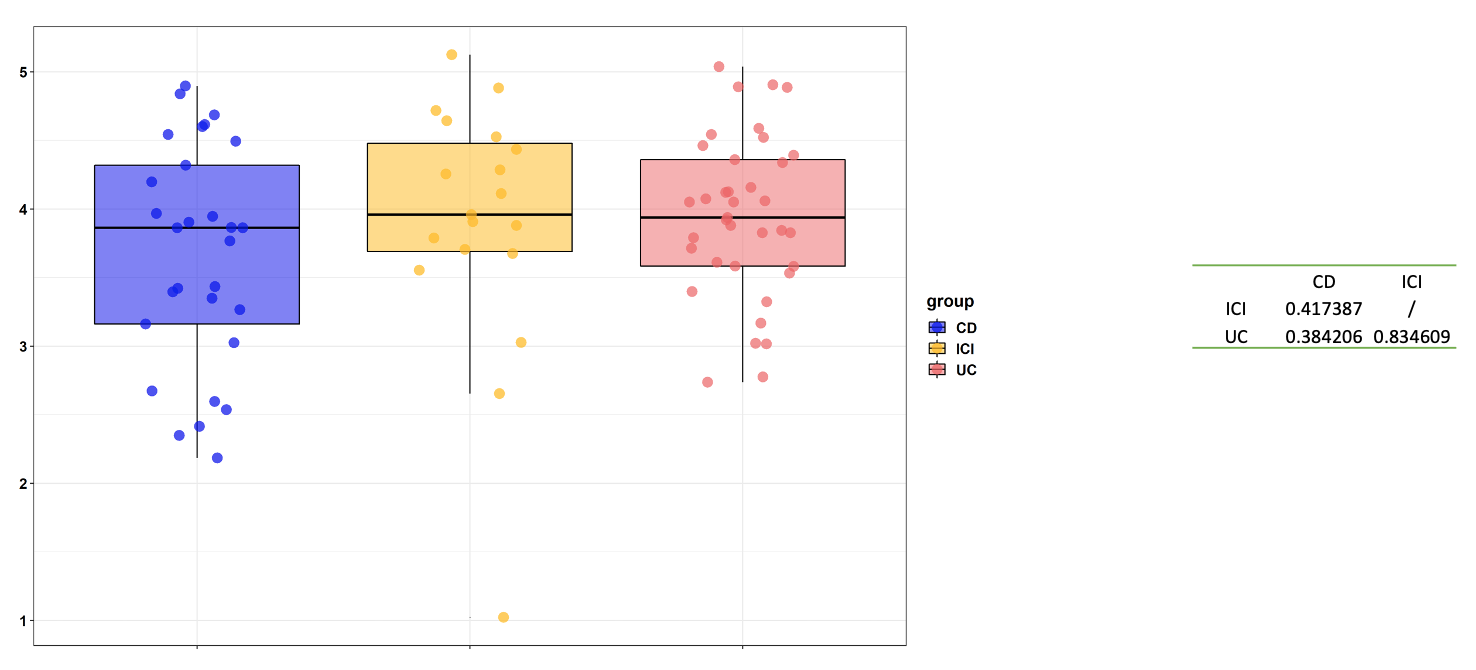


**C.**


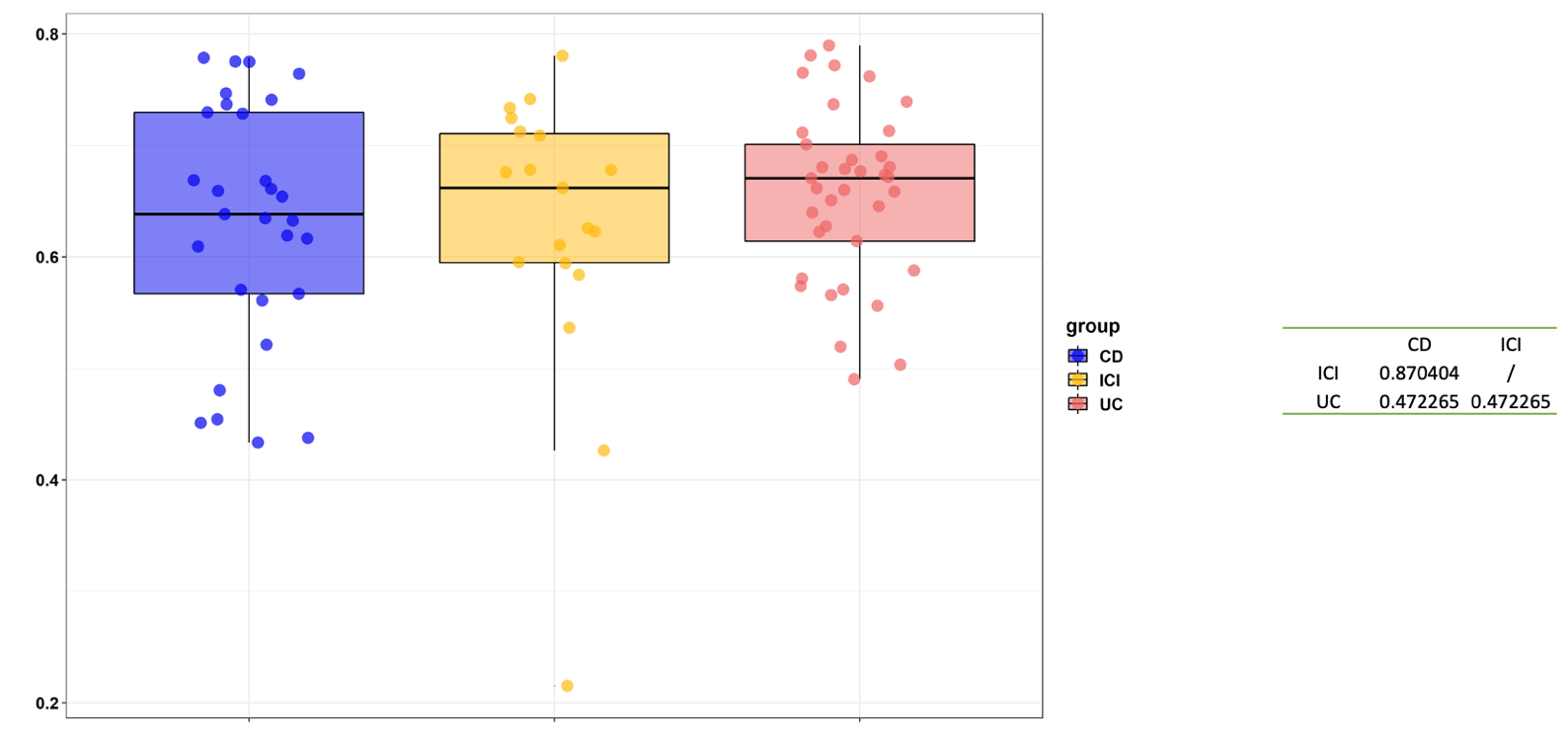


**Supplementary Figure 3. Alpha diversity analysis results for richness (A), Shannon (B), and Pielou (C) indices in patients with UC and IBD-like ICIs-colitis.**

**A.**


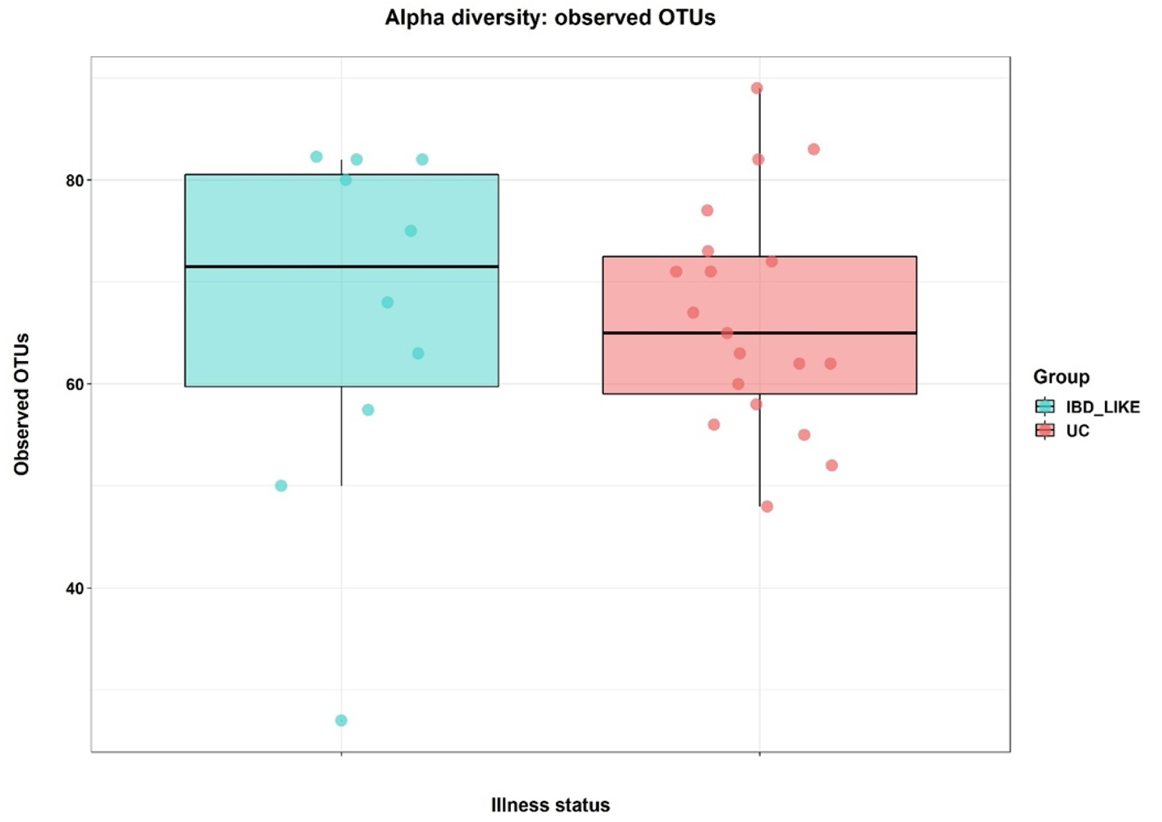


**B.**


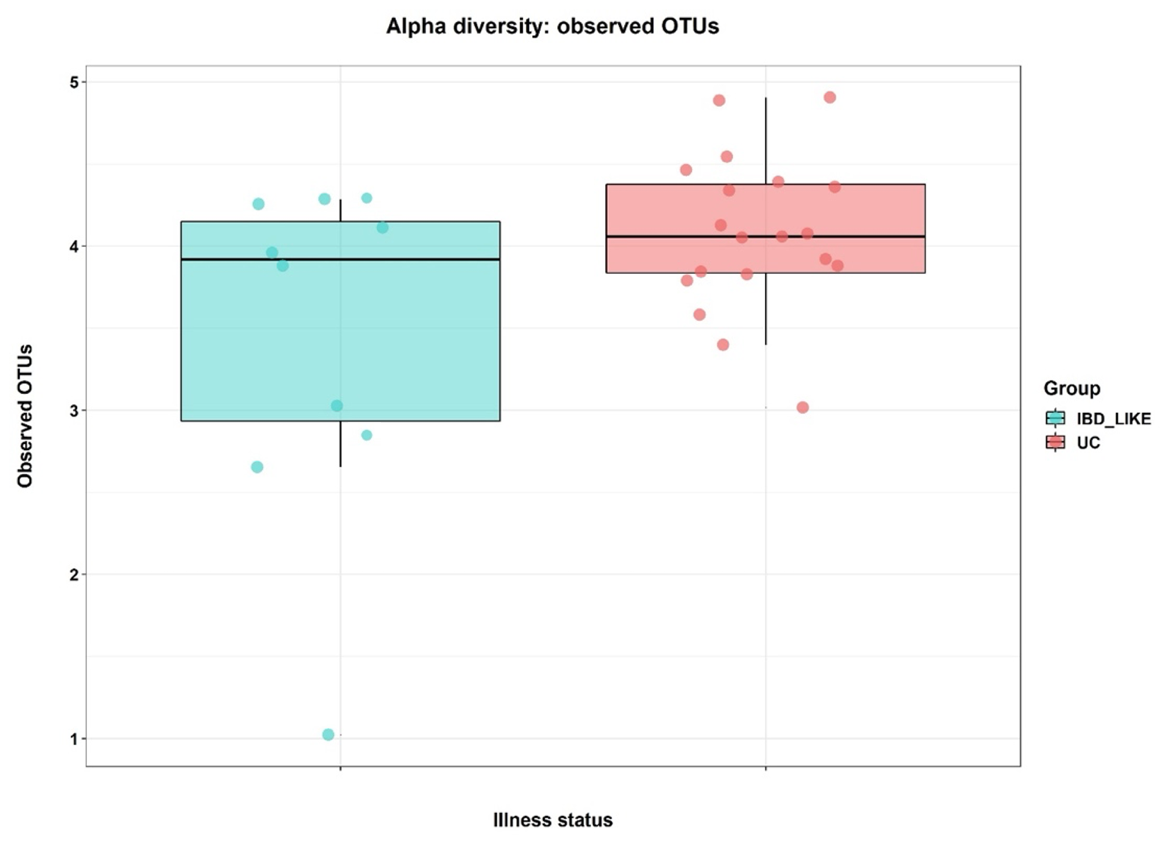


**C.**


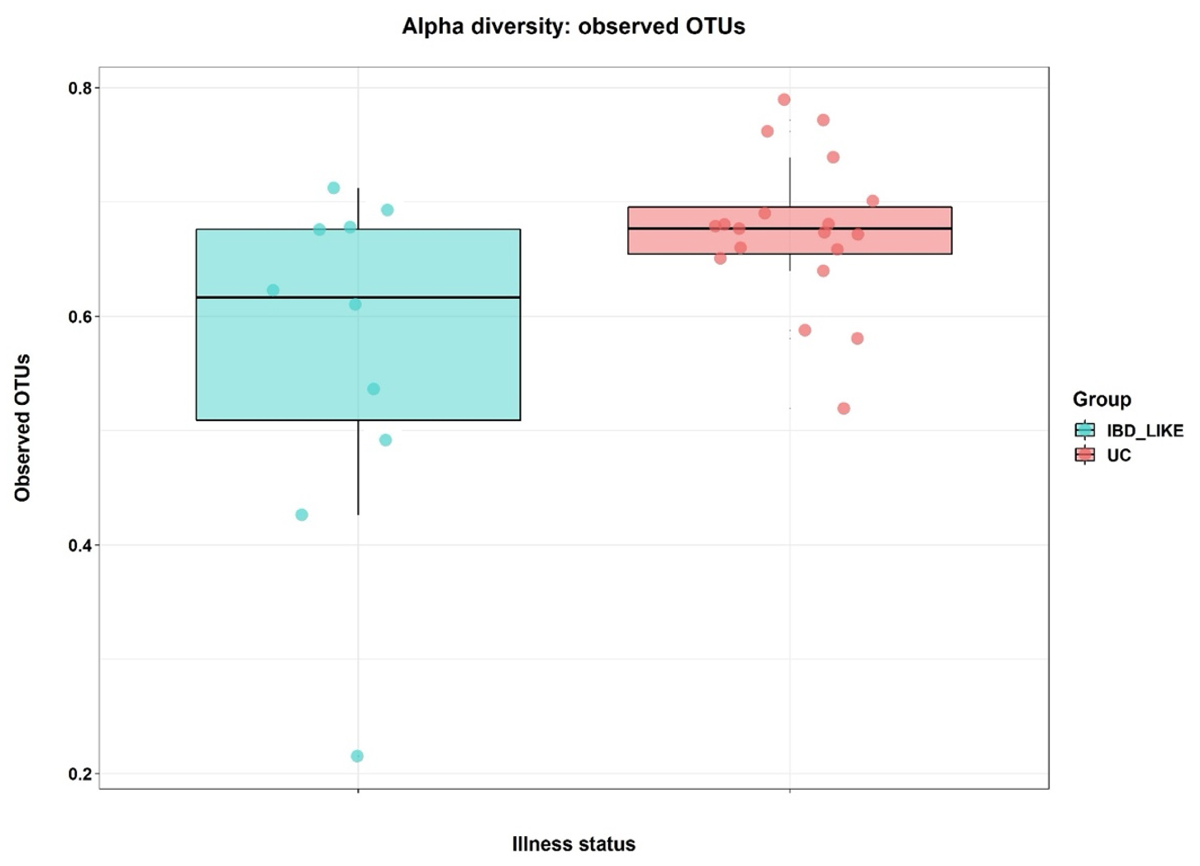


**Supplementary Figure 4. Non metric multidimensional scaling (NMDS) plot of Beta diversity (Bray-Curtis distance matrix) between healthy controls, patients with ICI-colitis, patients with active (moderate to severe) and inactive (in remission or mild) UC and CD.**


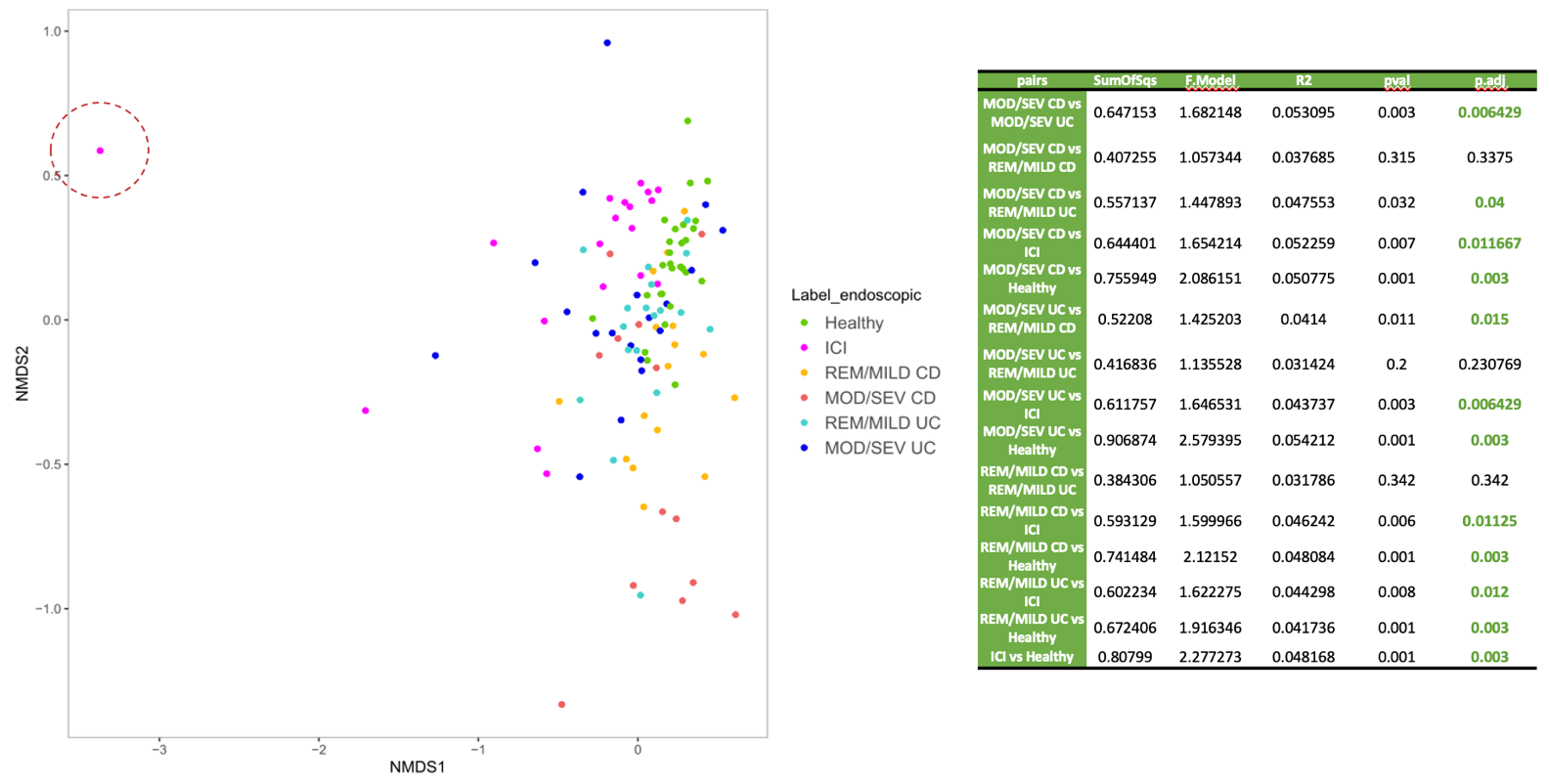


**Supplementary Figure 5. Non metric multidimensional scaling (NMDS) plot of Beta diversity (Bray-Curtis distance matrix) between patients with IBD-like ICIs-colitis and patients with UC.**

**
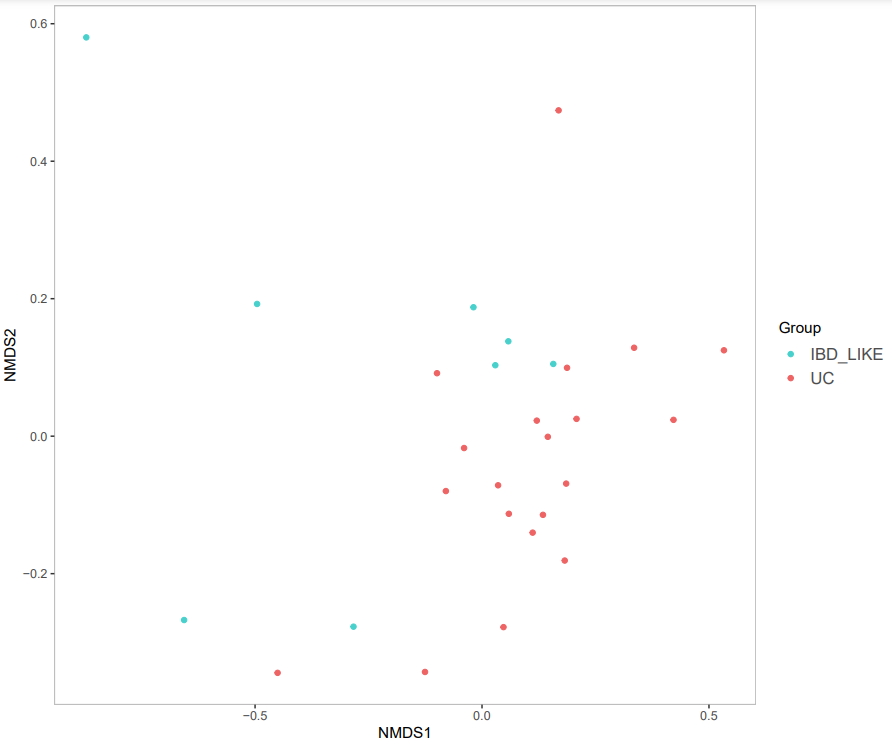
**

**Supplementary Figure 6. Random Forest confusion matrix. The training test was composed by 73 patients evenly chosen among the three groups. Data coming from the remaining 19 patients were used as test set for performance evaluation.**


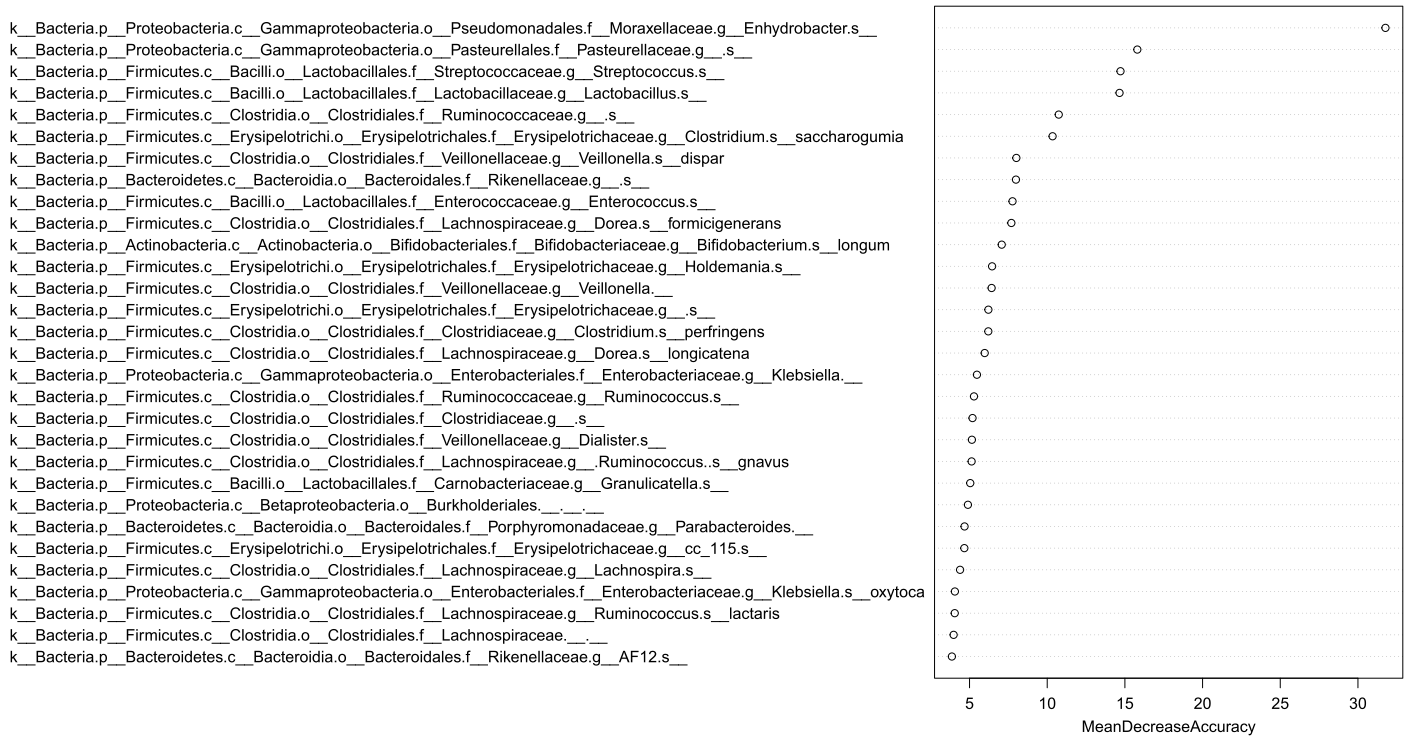


| **Predicted classes** | **CD** | **ICI** | **UC** |
| --- | --- | --- | --- |
| **CD** | 4 | 1 | 0 |
| **ICI** | 0 | 3 | 1 |
| **UC** | 3 | 0 | 7 |

Accuracy: 0.7368

95% CI: (0.488, 0.9085)

No Information Rate: 0.4211

p-Value [Acc > NIR]: 0.005381

Kappa: 0.587
